# Controlled communication between physically separated bacterial populations in a microfluidic device

**DOI:** 10.1038/s42003-018-0102-y

**Published:** 2018-07-20

**Authors:** Ekaterina Osmekhina, Christopher Jonkergouw, Georg Schmidt, Farzin Jahangiri, Ville Jokinen, Sami Franssila, Markus B. Linder

**Affiliations:** 1 Department of Bioproducts and Biosystems, School of Chemical Engineering, Aalto University, 02150 Espoo, Finland; 2 Department of Chemistry and Materials Science, School of Chemical Engineering, 02150 Espoo, Finland

## Abstract

The engineering of microbial systems increasingly strives to achieve a co-existence and co-functioning of different populations. By creating interactions, one can utilize combinations of cells where each population has a specialized function, such as regulation or sharing of metabolic burden. Here we describe a microfluidic system that enables long-term and independent growth of fixed and distinctly separate microbial populations, while allowing communication through a thin nano-cellulose filter. Using quorum-sensing signaling, we can couple the populations and show that this leads to a rapid and stable connection over long periods of time. We continue to show that this control over communication can be utilized to drive nonlinear responses. The coupling of separate populations, standardized interaction, and context-independent function lay the foundation for the construction of increasingly complex community-wide dynamic genetic regulatory mechanisms.

## Abstract (web summary)

Ekaterina Osmekhina et al. report a microfluidic device that allows complete control over growth and communication via quorum sensing between bacterial populations separated by a thin nano-cellulose filter. This enables the functionalization of multicellular populations, a strategy that could be used to construct complex genetic regulatory mechanisms

## Introduction

Development of highly complex, engineered synthetic biological systems requires combinations of different cell populations that together form communities. Each population can contribute with specific functions and they should function together in a synchronized manner. Inspiration is drawn from complex naturally occurring multicellular networks such as the microbiota^1^ and the plant root rhizosphere^2^ where the complexity and diversity of functions performed by the community far surpasses the capabilities of individual cells. Cooperation between cells reduces the individual metabolic burden and enables the specialization of populations^3–7^. Finding means for utilizing controlled cellular communication is critical.

A much-desired feature when working with multicellular communities, is the context-independent control over the individuals within the community^8–12^. In this, populations can be programmed or chosen based on a specialized or desired function, regardless of the other populations^13,14^. Synchronizing the function of these programmed populations through well-controlled interactions could enable the construction of more complex community-wide regulatory systems and more efficient expression of metabolic networks^15,16^.

In reality, however, cellular communication is very challenging to control and current efforts to functionalize or program populations as parts of communities face difficulties in their setup. When not using physical separation, i.e., using co-cultures, an overgrowth of the community by a single population often occurs^3,6,14^. The overgrowth makes it hard to study individual cellular interactions, and hence also communication and expression. Attempts to prevent overgrowth have led to the design of cellular interdependencies, in which individuals produce essential metabolites necessary for the survival or growth of the other^5,17–19^. However, these interdependencies are complicated to construct, can lead to a high metabolic burden, may interfere with other pathways, and show fluctuations of relative population sizes in the community.

An alternative strategy is to use physical barriers to separate cells, with an example being the use of polycarbonate membranes in microfluidic devices^18^. However, setups used so far drastically reduce the efficiency of communication, delaying the responses of populations and cause a break in synchrony. These methods also face uneven growth and variations in expression rates, making the communication highly variable and time-dependent, as they have been difficult to implement as continuous systems^18,20,21^. Chitosan has also been used to construct membranes within microfluidic devices to separate cellular populations^11,12^. Because of the architecture of the system however, communication is established on the timescale of hours. This makes it not ideal for rapid and repeated on and off switching of genetic elements in a population. Thus, a much needed tool in developing cellular communication within communities is a system in which the stable and independent function of each population can be studied and controlled over long periods of time, similar to the function of individual processors on a circuit board^21^.

Here we present a method that enables long-term stability and control over cellular growth, communication, and gene expression in two separate and independently growing populations. We demonstrate how cellular communication leads to synchronized community-wide functions, both through external induction and by dynamic feedback loops.

## Results

### Construction of the microfluidic device

**Fig. 1:**
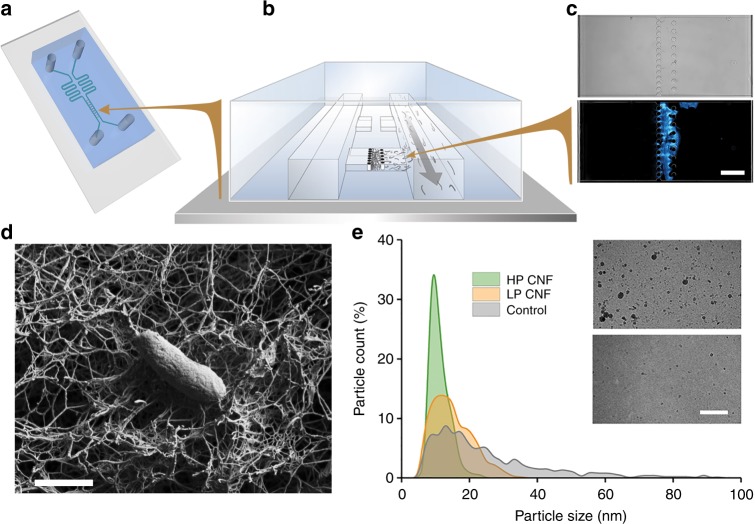
Microfluidic device that enables communication between *E. coli* populations through a CNF filter. **a** PDMS microfluidic chip on a glass slide. **b** Loading of CNF into the traps through the channel. CNF entangles between the rows of pillars. **c** Transparent light (upper) and fluorescent (lower) images of a CNF filter stained with calcofluor white. Scale bar, 30 µm. **d** Representative SEM image of a CNF network upon which an *E. coli* cell was placed, illustrating that the pores in the highly entangled CNF fibers are too small for cells to penetrate. Scale bar, 800 nm. **e** Analysis of the size distribution of nanoparticles show the porosity of CNF filters, where the high-pressure compressed (HP) filters, as seen in the bottom TEM image, allow passage of particles with a diameter of maximally 24 nm. Filters made by compression under low pressure (LP), as seen in the top TEM image, allow the passage of larger particles. The larger pore sizes led to penetration of the CNF filter by bacterial cells over time. Scale bar, 200 nm

We constructed a polydimethylsiloxane (PDMS)-based microfluidic device, in which the growth of cells takes place in, and is confined to, a trapping chamber. This trapping chamber is divided into two parts by a filter made of rows of PDMS pillars and cellulose nanofibrils (CNFs)^22^ entangled between them (Fig. 1a–d, Supplementary Fig. 1 and 2). The CNF layer functions as a filter separating two bacterial populations while allowing efficient signal exchange between them. Both sides of the chamber are connected to separate channels that run parallel to each other and are used for steady nutrient supply to ensure continuous cellular growth. Because of the confined geometry of the growth chambers, both populations contain equal numbers of bacterial cells, where excess cells automatically get pushed into the main channel and removed by the high flow rate.

CNF proved to function ideally as the filter material for dividing the communicating traps. The basis of the function of CNFs for building a filter are their physical properties. Individual CNF fibrils consist of a crystalline arrangement of cellulose molecules packed unidirectionally. CNF fibrils have a diameter of about 5 nm and lengths of several tens of micrometers. The CNF used here was obtained by highly disintegrating birch pulp and therefore have a surface coating of hemicellulose, mainly xylan^23^. They are thus long and stiff colloidal objects that interact weakly with each other. In a dilute solution they can flow freely, but if they encounter an obstacle, such as the pillars between parts of the trapping chamber, they become physically entangled. The entanglement of several fibrils creates multiple interactions and together they form a network. Such a high concentration network will be highly entangled and have gel-like properties. The gel then functions efficiently as a filter, as the network formed by the stiff fibrils of CNF contains voids on the sub-micrometer scale, and is physically very stable^22^. The entangled networks formed in our microfluidic devices had pores considerably smaller than the size of individual bacteria. Figure 1d shows an individual *Escherichia coli* cell on a cellulose network, which was made outside the microfluidic device to illustrate proportions of the cellulose fibers, the porosity of the filter, and a bacterial cell (cellulose porosity measurements are shown in Supplementary Fig. 3). In Supplementary Fig. 4c, it is shown that depending on how the cellulose filter was prepared, the pore-size of the membranes could be adjusted. The CNF network remained porous enough to allow liquid and small molecules such as acyl-homoserine lactone (AHL) to pass through it. The surface of the cellulose is inert and it showed no detectable interactions with cells nor with AHL (Supplementary Fig. 5). As CNF was applied as a suspension to the preassembled PDMS devices, the filters could easily be placed at multiple selected locations simultaneously (Supplementary Fig. 1).

Two parameters had a clear effect on the formation of an efficient CNF filter in the trap. First, the gaps between the pillars of solid PDMS required optimization. We found that a single row of pillars with gaps of 2 µm in between resulted in well-functioning filters (Fig. 1b, c, Supplementary Fig. 2). With 5 µm gaps, individual CNF fibrils were not sufficiently interlocked and were washed through into the other channel. With 1 µm gaps, the flow between the two sides of the trap was insufficient. A second row of more widely separated pillars (10 µm) was used to further keep the CNF filter in place (Supplementary Fig. 2). Second, the CNF filter had to be optimally packed by applying increased flow (Fig. 1c–e, Supplementary Fig. 1). The CNF solution was initially applied at low velocity through a single channel, allowing entanglement and trapping of a thick layer at the pillars. Next, the CNF layer was gradually compressed at either low pressure (LP, by flow in the main channel at 0.13 m s^−1^) or high pressure (HP, by flow in the main channel at 1.28 m s^−1^). To assess the porosity of filters packed at the different pressures, we used polydispersed silicon oxide nanoparticles (10–100 nm) and analyzed their composition by cryogenic transmission electron microscopy (CRYO-TEM), before and after flowing through the filter in the device (Fig. 1e, Supplementary Fig. 3). LP-packing resulted in entrapment of particles larger than 42 nm and also allowed bacterial cells to pass through the filter over time (Supplementary Fig. 4a). HP-packing led to entrapment of particles above 24 nm and proved suitable in keeping bacterial cells separated over long periods of time (Supplementary Fig. 4b). In the case of insufficient packing, a single population moved over to the other side, resulting in overgrowth of the entire trap within a few hours, as seen in Supplementary Fig. 4a.

### Stable and dynamic communication between populations

**Fig. 2:**
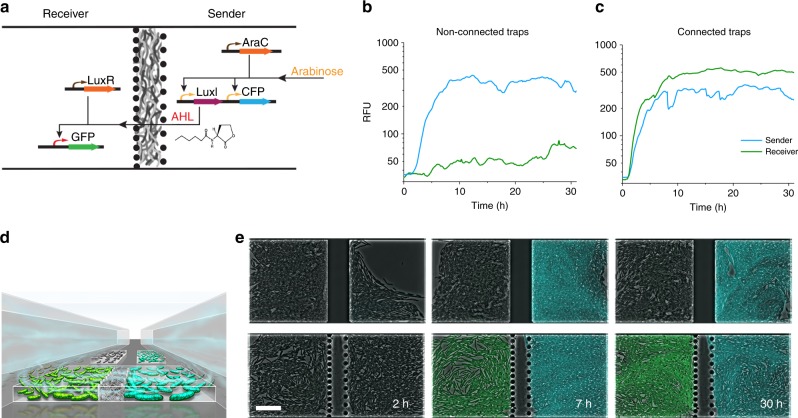
Stable communication through a CNF Filter. **a** Genetic circuit design. In the sender cells on the right side of the trap, an arabinose-inducible promoter drives production of CFP and also LuxI, which enzymatically synthesize AHL. AHL diffuses through the cell membrane and the CNF filter to the left side of the trap, and into the receiving cells, where, together with the constitutively expressed LuxR, it induces the expression of GFP. **b** Mean fluorescence intensities in the chambers as a function of time for the non-connected and **c** connected trapping chambers. Blue line, intensity of CFP of the sender cells. Green line, intensity of GFP of the receiver cells. The y axis has a logarithmic scale. **d** Schematic of a trap with loaded cells. **e** Phase contrast merged with fluorescent images of the populations in the microfluidic traps during a long-term experiment illustrating stable communication. The sender cells were induced with arabinose after 3 h of growth inside the chambers. In the connected chambers, the receiver population responded to the AHL produced by the sender cells and generated a green fluorescence. In the non-connected chambers, the receiver cells stayed dark as they did not receive AHL. Scale bar, 30 µm

To demonstrate the communication of bacteria physically separated by the CNF filter, we designed two populations of *E. coli* cells termed sender and receiver (Fig. 2a–e). The cells were loaded through parallel channels in the microfluidic chip, each colonizing opposite sides of the chamber and separated by the filter. The sender cells produced a cyan fluorescent protein (CFP) and the Lux AHL in response to induction with arabinose. The Lux AHL was originally identified as part of the quorum-sensing mechanism of *Aliivibrio fischeri* and is a small molecule that is able to freely move through cell membranes and mediate cell communication^24^. Once produced by the sender cells, the AHL diffused through the CNF filter to the receiver population and induced the expression of green fluorescent protein (GFP) as a response. The lack of a visible delay between the generation of the GFP signal compared to the CFP signal indicated that on the timescale of the experiments there was a free, unhindered movement of the AHL from one side to the other in the microfluidic trap (Fig. 2c, Supplementary Movie 1). In a negative control, the CNF filter was replaced by a solid block of PDMS, preventing the sender and receiver populations from communicating. As a result, the AHL diffusion was hindered and the receiver cells stayed uninduced. We observed stable cell growth and signal generation in both populations over 30 h, during which the CNF filter remained impermeable to cells.

**Fig. 3:**
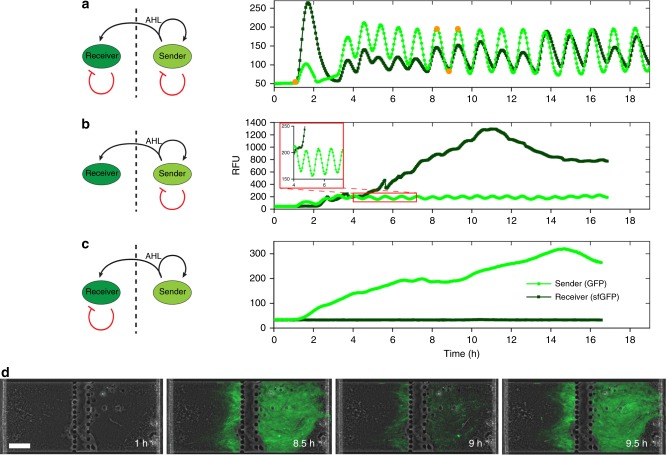
Synchronized oscillations in communicating populations. **a** Oscillations with negative feedback loops, induced by the AiiA lactonase in both sender and receiver populations. In the sender population, expression of the luxI, aiiA, and gfp genes is controlled by the lux promoter. LuxI enzymatically produces AHL, which activates the lux promoter. The AHL lactonase, AiiA, hydrolyzes AHL, providing the negative feedback. AHL diffuses into the receiver cells and induce production of sfGFP and AiiA. Mean fluorescence intensities in the traps as a function of time illustrate the oscillations. The light green curve shows oscillations of GFP in the sender cells, whereas the dark green curve shows oscillations of sfGFP in the receiver cells. **b** Absence of aiiA (negative feedback) in the receiver population resulted in the accumulation of sfGFP. **c** Absence of aiiA in the sender population resulted in the accumulation of the fluorescent protein, whereas in the receiver population, the continuous induction of aiiA resulted in the complete inhibition of the sfGFP production. **d** Phase contrast merged with fluorescent images of the cell populations in the microfluidic traps during the experiment described in (**a**), illustrating a dynamic communication at different phases of the oscillations. Time points at which the images were collected are shown in curve (**a**) as orange dots. Scale bar, 30 µm. In **a**, **b**, and **c**, the fluorescence intensity was collected from images taken of traps every third minute. Individual data points are shown as dots with connecting lines

We proceeded to demonstrate a dynamic communication through the CNF filter, where we studied the role of positive and negative feedback loops to generate stable oscillations (Fig. 3). The senders utilized a genetic network based on quorum-sensing elements from *A. fischeri* and *Bacillus thuringiensis* originally developed by Danino et al.^25^. Three genes of the network, *luxI*, *aiiA*, and *gfp*, are under the control of the same *lux* promoter (*pLux*). LuxI produces AHL, which activates the transcription of all these genes in a complex with LuxR. The lactonase aiiA degrades intracellular AHL and, as a consequence, reduces *lux* promoter activity. The combination of positive and negative feedback loops leads to the oscillating production of AHL and visualized by GFP expression. This population-wide synchronized oscillation had a period of about 1 h. In our microfluidic communication system, the expression waves of AHL from the sender cells were detected by the receiver population, where the fluorescent signal followed the oscillations seen in the sender population (Fig.3 a, d, Supplementary Movie 2).

Initial attempts to achieve coupled oscillations in the receiver population resulted only in a weak fluorescence response. This was likely due to the diffusion distance, which in combination with degradation of AHL by lactonase, prevented sufficient accumulation of AHL. Therefore, a brighter and fast-folding form of GFP, sfGFP, was used in the receiver cells, which resulted in detectable signals. When responding, the initial peak of sfGFP intensity in the receiver cells was clearly higher than subsequent ones, likely due to the absence of LuxI synthase in the receiver cells. The lack of a synthase in the receiving cells prevented low levels of *aiiA* expression during the early stages of growth, when cells divided and filled up the trapping chambers. These were only activated when a quorum was reached in the sender cells, resulting in a strong expression of both *sfGFP* and *aiiA* in the receiver cell*s*. A negative feedback in both sender and receiver proved crucial in generating stable oscillations. When a single negative feedback was present in the sender populations, but absent in the receiver population, the fluorescent signal accumulated to very high levels (Fig. 3b, Supplementary Fig. 6b). The receiver population showed increases in fluorescent signal that coincided precisely with the increase of GFP fluorescence in the oscillating sender population. On the other hand, when the negative feedback *(aiiA*) was only present in the receiver population, no fluorescent signal could be detected, whereas the sender population showed strong signal accumulation (Fig. 3c, Supplementary Fig. 6c). Because of the absence of a negative feedback loop, and as a result the continuous production of AHL, an increasing buildup of fluorescence was seen in the responding population, when compared with the receiver population of Fig. 3b.

### Synchronization of coupled oscillations

**Fig. 4:**
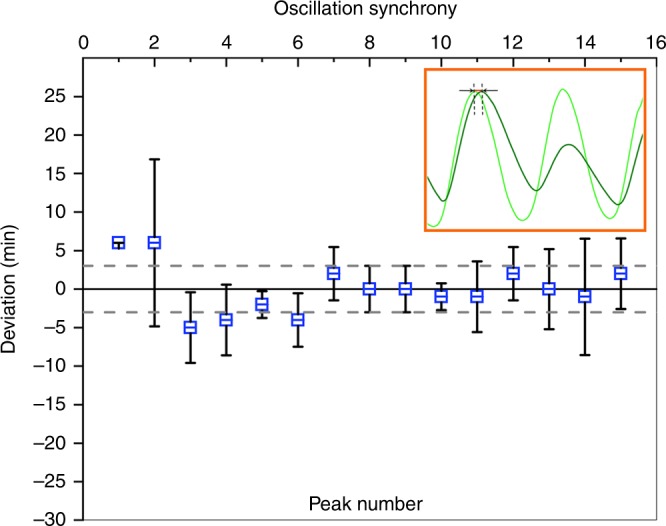
The synchronization of coupled oscillations in connected populations. The average response time of the fluorescent signal peak in the responder population (dark green, sfGFP) was compared with the oscillating sender population (light green, GFP) (as in Fig. 3a). Data from three separate traps

To characterize the coupling delay, we analyzed the synchrony in oscillations between sender and receiver (Fig. 4). After the initial burst of sfGFP, the secondary oscillations in the receiver cells closely followed the primary oscillations in the sender cells. Analysis of the deviation between sender and receiver was measured every third minute and gave no apparent delay in response, showing that the diffusion of AHL across the filter, and synchronization of the populations, occurred rapidly. This was in line with our calculations of AHL diffusion^26^, showing that quorum-sensing-based signaling is an excellent way to couple community-wide behavior that acts much faster, by estimation in less than a minute (Supplementary Fig.7), when compared with transcription factor and expression-based coupling (~30 min)^27^. The combination of rapid quorum-sensing-based coupling together with the slower transcription factor-based coupling could provide an interesting tool to control time-dependent nonlinear responses in individual populations.

## Discussion

Here we have presented a method that enables the independent growth of two separated cellular populations, where the number of cells and growth state remains stable over long periods of time. A thin CNF filter allows rapid communication, with diffusion times of around 1 min, between the populations through diffusion of AHL as a signaling molecule, and prevents uncontrollable growth of a single population. This results in modular control over individual populations, where each population provides a predictable output in protein expression (either on or off) that is based on its input. In experiments, output or “performance” is measured by fluorescence intensity. This can both be externally controlled using arabinose and internally controlled using genetic circuit design. This allows multicellular strategies to divide complex circuits over multiple populations, dividing the genetic burden on each individual.

Potential benefits of such an approach are in synthetic biology setups where genetic circuits are built to perform logic operations^13^. Logic gates can be constructed in bacteria using sets of genetic regulatory elements. To combine several gates within only one type of cell is generally technically challenging, partly because of the limited amount of regulatory elements that are available and suitable for building logic gates, and the overall expression burden on the bacterial host^3^. One problem is that the parts must be orthogonal, i.e., not interfering with other parts within a cell. Hence, wiring different cell types together is essential for building more complex systems, as the same part can be reused if separated into different cells. As such wiring between cell types can be done using signaling molecules such as AHLs, the dynamic communication shown in this study could lead to more efficient and responsive wiring, reduced individual burden, and hence better performing systems^3,28,29^.

Another benefit of studying the dynamics of gene expression is that it can reveal new basic information on mechanisms of gene transcription and regulation in cells^30^. Such dynamic responses and cellular communication has for example been used with microbial cells to create patterns or structures by signaling interactions. This was demonstrated by genetic programming to produce stripe patterns in expanding cell populations by achieving variable cell densities on a solid growth medium^31^. We suggest that the coupling of different populations by dynamic signaling interactions could lead to new approaches for understanding pattern formation in biology, using microfluidic devices as described here as a basis.

Another possible application lies in the screening of compounds that could influence the quorum-sensing communication. Many pathogenic Gram-negative bacteria produce quorum-sensing molecules and in many cases, such as *Pseudomonas aeruginosa* and *Acenitobacter baumannii*, the quorum-sensing molecules have been directly linked to their pathogenesis^32,33^. This has resulted in large interest for compounds that can directly target these quorum-sensing molecules, termed as quorum quenching^34^. The microfluidic setup enables detailed information about the efficacy of a particular quorum quenching compound, the speed at which it has an effect, and the possible side effects of the compound on the bacterial cells, in real time.

The continuous function of our system lends itself particularly well to study dynamic events, as demonstrated here by coupling an oscillatory function, which showed repeated switching of states between on and off. Such long-term dynamic communication capabilities resemble natural communities more closely, where its individuals continually act and react on one another. These communities, such as the plant rhizosphere^2^ and gut^1^, are often highly sensitive, complex and difficult to study^18^. The controlled environment in the trapping chambers could help to understand, and in the future also possibly utilize, the individual cellular populations that operate in a controlled community.

## Methods

### Strains and growth conditions

The *E. coli* strains MG1655, CY008 (BW25113 ΔlacI ΔaraC ΔsdiA), and Top10 were used in this study. Individual colonies of each bacterial strain were cultured overnight in Luria Broth (LB) medium with the appropriate antibiotics, ampicillin (50 μg mL^−1^) and kanamycin (30 μg mL^−1^), at 37 °C. The next day, cultures were diluted 1:1000 in 2 mL of fresh medium and grown to 0.2 OD_600_. Afterwards, cells were spun down, resuspended in 50 µL LB, and loaded into the microfluidic channels.

### Plasmid construction

Individual fragments were amplified using High Fidelty PCR Genestrands (Eurofins Genomics GMBH). Plasmids were assembled with Golden Gate restriction digests^35^. Fragments were cloned into two different vector backbones, namely pACYC (amp) for the receptor proteins and AiiA lactonase, and pColE1 (kan) for the AHL synthases and fluorescent proteins. The plasmids were transformed into Top10 and verified through DNA sequencing. As fluorescent proteins, CFP^36^ (Y66W/F145Y/N146Y/H148D derivative of sfGFP), sfGFP^37^, and GFP were used. Synthases and fluorescent proteins were tagged with LAA sequence, targeting them for protein degradation through the ClpXP degradation system^38^.

### Fluorescence microscopy

Phase contrast and fluorescent images were acquired using an Axio Observer Z1 microscope (Carl Zeiss, Jena, Germany) at × 20 magnification every 6 min for stable communication experiments and every 3 min for the coupled oscillation experiments. The focus was maintained automatically using the definite focus option of the microscope. During the experiments, the microfluidic chip temperature was maintained at 37 °C and the media flow rate was kept at 300 µm s^−1^ in both main channels using an automated syringe pump. The CNF (0.5 % solution) was loaded before each experiment and compressed with a flow rate of 1.28 m s^−1^ (Supplementary Fig. 1). CFP signal was obtained using excitation light at 420 nm, while collecting the emitted light of 461–485 nm. GFP signal was obtained using excitation light at 480 nm, while collecting the emitted light of 515–535 nm.

The mean intensities of the fluorescent light (relative fluorescent units) were measured over the trap areas for each acquisition point using ZEN software (Carl Zeiss). The data were plotted against the time and the measured points were connected with straight lines (no curve fitting was used).

### Preparation of nanofibrilated cellulose

Hardwood birch pulp, obtained from the Finnish pulp mill (UPM, kappa number 1; DP 4700; fines removed SCAN-M 6:69) was used to produce the CNFs by fluidizing never-dried fully bleached sulfite hardwood. A high-pressure fluidizer (Microfluidics M110P, Microfluidics Int. Co., Newton, MA) was used to disintegrate the wood pulp, passing six times through the fluidizer to decrease the fiber size^39^. No chemical or enzymatic pre-treatment was used before disintegration. The cellulose was autoclaved and stored at 4 °C. Before use, cellulose was diluted to a 0.5% w/v concentration with distilled H_2_O. This solution was homogenized with a Polytron Homogenizer PT 3100 D (Kinematica AG, Switzerland) at 20,000 r.p.m. for 3 min and centrifuged at 5000 r.p.m. for 5 min, to remove large cellulose fibers, after which the supernatant was applied in the syringe to load in the microfluidic chips.

### Confocal microscopy

Calcofluor white (Sigma-Aldrich) was used to fluorescently stain cellulose. One drop of calcofluor white was applied together with one drop of 10% potassium hydroxide and added to the medium, staining the cellulose in the microfluidic traps. Confocal micrograph stacks were acquired with a Zeiss LSM 710 microscope (Carl Zeiss) with either a × 40, 1.6 numerical aperture (NA) oil-immersion objective or a × 20, 1.6 NA air phase-contrast objective using excitation light at 405 nm and collecting the emitted light from 410 to 580 nm.

### Electron microscopy

Droplets of CNF solution were pipetted into a 1:1 mixture of propane and ethane liquid at − 196 °C, to ensure rapid freezing and to prevent the collapse of cellulose fibrils. Bacterial cells were dehydrated with repeated washing of 2.5% glutaraldehyde and increasing concentrations of ethanol (50%, 70%, 80%, and 90%), after which samples were air dried. Platinum sputtering was used to apply a 5 nm coating to the samples (Emitech K590X/K350) before imaging^40^. Scanning electron microscopy was performed using a Zeiss Sigma VP (Carl Zeiss) with an in-column SE detector and an accelerating voltage of 1 kV.

### Cellulose porosity measurement

Polydispersed silicon oxide nanoparticles were purchased from Iolitec (Heilbronn, Germany). A mixture of the polydispersed silicon nanoparticles (ranging from 10 to 100 nm) with a concentration of 0.1% w/v was treated with a Q500 ultrasonic tip sonicator (Qsonica, USA), at 25% amplitude for 20 min and 400 W power output, to break particle agglomerates and achieve particle dispersion. The nanoparticle solution was applied into the microfluidic chip preloaded with CNF. The supernatant was collected from the other channel, i.e., fluid that passed through the filter, which was directly applied on a carbon grid and analyzed with a FEI Tecnai 12Bio Twin transmission electron microscope (Hillsboro, USA). Analysis of the particle size distribution was performed using ImageJ software^41^.

### Microfluidic fabrication

The microfluidic chip was designed and fabricated by standard soft lithography and replica molding approach^42^. Initially, a master mold including SU-8 (MicroResist GmbH) on silicone wafer was created by spin-coating two distinct layers, SU-8-5 for the 1.6 µm layer and SU-8-50 for the 20 µm layer, respectively. Each layer was exposed with a mercury lamp i-line (3 and 8.5 s, respectively). After development of the topographies, the surface was coated with a ~20 nm fluorocarbon polymer film. PDMS was prepared by mixing of monomer and crosslinking agent in 10:1 ratio (Sylgard 184 kit, Dow Corning), degassed, and cast on the microfluidic mold, followed by an overnight curing step at 70 °C. Afterwards, individual PDMS chips were cut and punched (input and output holes), and cleaned using a 2 h n-pentane wash followed by a 1 h acetone wash. Chips were air dried, after which they were bonded to glass coverslips by oxygen plasma treatment. An overview of the microfluidic chip design is shown in Supplementary Fig. 8.

### Data availability

Data sets acquired in this study are available^43^ for download at Zenodo 10.5281/zenodo.1297361. This data set contains processed movies of both stable and oscillating communication, data points from which fluorescent images are obtained, as well as the oscillation peak analysis and the CNF porosity measurement using nanoparticles.

## Electronic supplementary material

Supplementary file

Description of Additional Supplementary Files

Supplementary Movie1

Supplementary Movie2
