# Supplementary material for: Controlled communication between physically separated bacterial populations in a microfluidic device
Source: Commun Biol. 2018 Jul 20;1:97. doi: 10.1038/s42003-018-0102-y (PMC6123784; doi:10.1038/s42003-018-0102-y)
Supplement: Supplementary file 1 — Supplementary file [file 42003_2018_102_MOESM1_ESM.pdf]

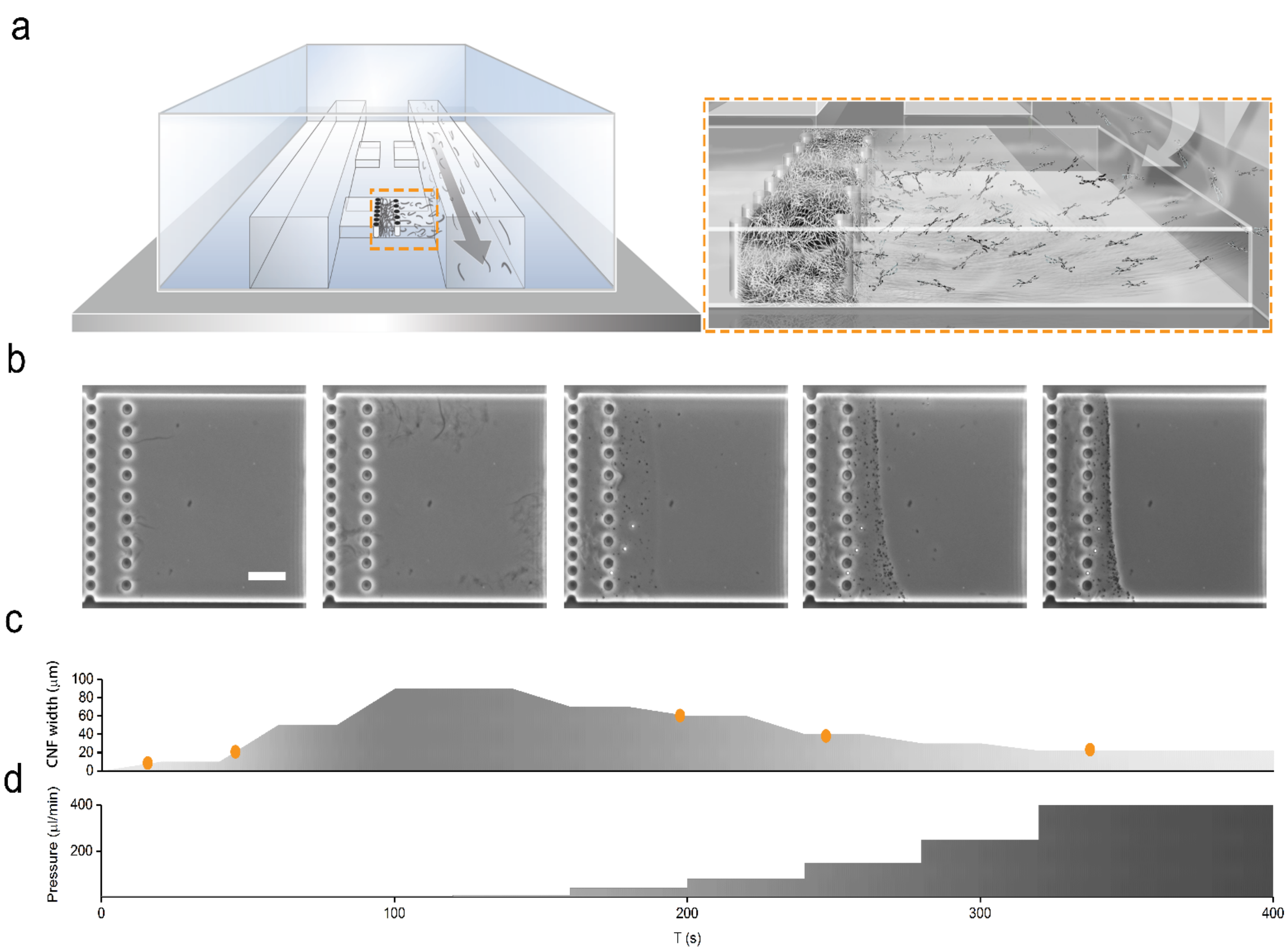

Supplementary Figure 1. Formation of the CNF membrane inside a microfluidic device. a) Schematic representation of the microfluidic channels and trapping chambers, where CNF applied in the right channel forms a porous membrane at the PDMS pillars. **b**) Phase contrast images of CNF in the trapping chamber during different stages (corresponding to the orange markers) of the porous membrane formation. Scale bar, 20 µm. **c**) Corresponding thickness of the CNF membrane during the preparation inside the trapping chambers. **d**) Flow rate profile. Low flow rates at the start gather a large amount of fibrils, after which gradual increases in flow rate, densely pack the membrane.

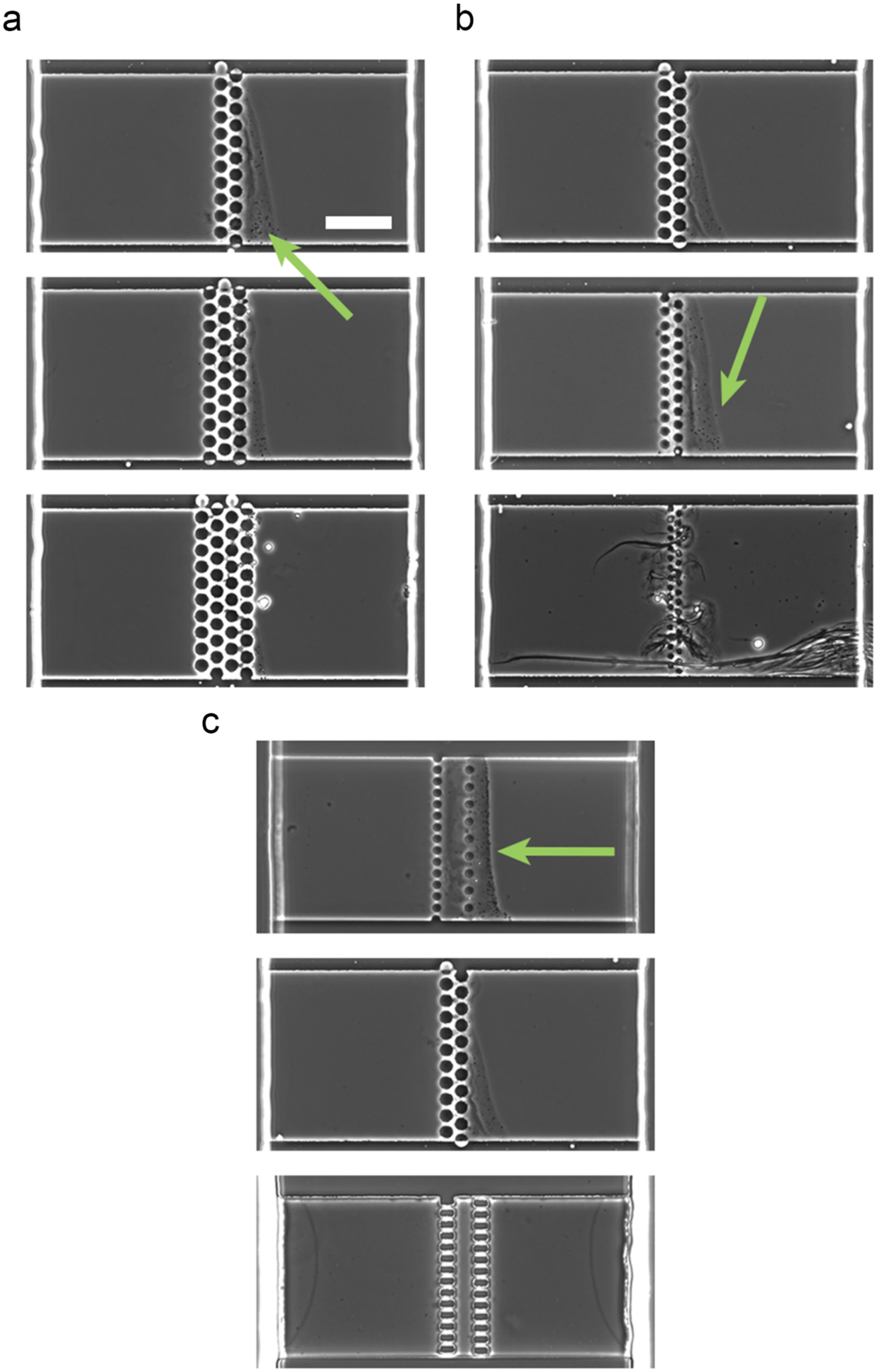
a
b
c

Supplementary Figure 2. Effect of the pillar sizes and arrangements on the CNF membrane formation. Phase contrast microscopy images of **a**) increasing number of pillar rows. Increasing rows reduced the flow-through of the CNF solution, which decreased the thickness of the formed membrane (forming on the right side of the pillars); **b**) varied pillar sizes (8, 4 and 2 µm respectively), where the 2 µm pillars faced uneven attachment to the glass surface, due to their small size; and **c**) varied geometries on the CNF membrane formation. Green arrows indicate the best parameter in regards to CNF membrane formation. Placing a first 2 µm pillar row, followed by a 5 µm spaced pillar row to keep the CNF membrane firmly in place, resulted in the most successful and densely packed membrane. Absence of the more widely spaced pillars allowed occasional movement of the CNF filter. Scale bar, 40 µm.

a

	Average distribution	Peak Distribution	Largest particle size	Smallest particle size
No CNF	26 nm	16-20 nm	94 nm	6 nm
Low Velocity	16 nm	16-20 nm	35 nm	6 nm
High Velocity	12 nm	11-15 nm	24 nm	7 nm

b

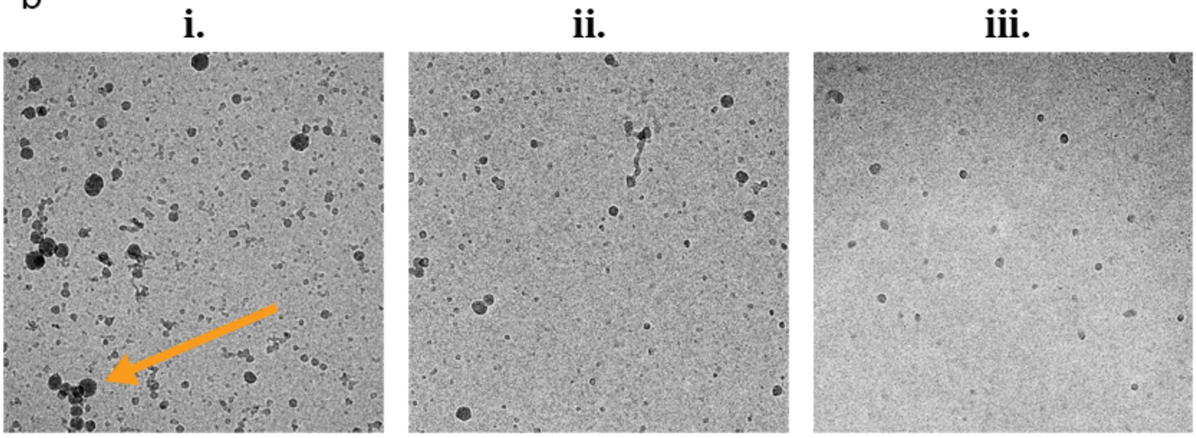

Supplementary Figure 3. Cellulose porosity measurements. Due to the placement of the CNF membrane inside a microfluidic chip, characterization of the membrane porosity is difficult using conventional methods such as thermoporometry. To accurately determine the porosity and the packing density of the CNF membranes formed at the PDMS pillars, we analyzed polydisperse nanoparticles passing through the filter using CRYO-TEM. **a**) Table highlighting key points from the nanoparticle size distribution analysis. **b**) CRYO-TEM images flow-through i. without CNF ii. Low velocity compressed CNF (0.13 m s^{-1} in the main channel) and iii. high velocity compressed CNF (1.28 m s^{-1} in the main channel). Although CRYO-TEM was used to prevent nanoparticle clustering, small clusters of particles still remained, as indicated by the orange arrow. These regions that were excluded in measurements. Scale bar, 200 nm.

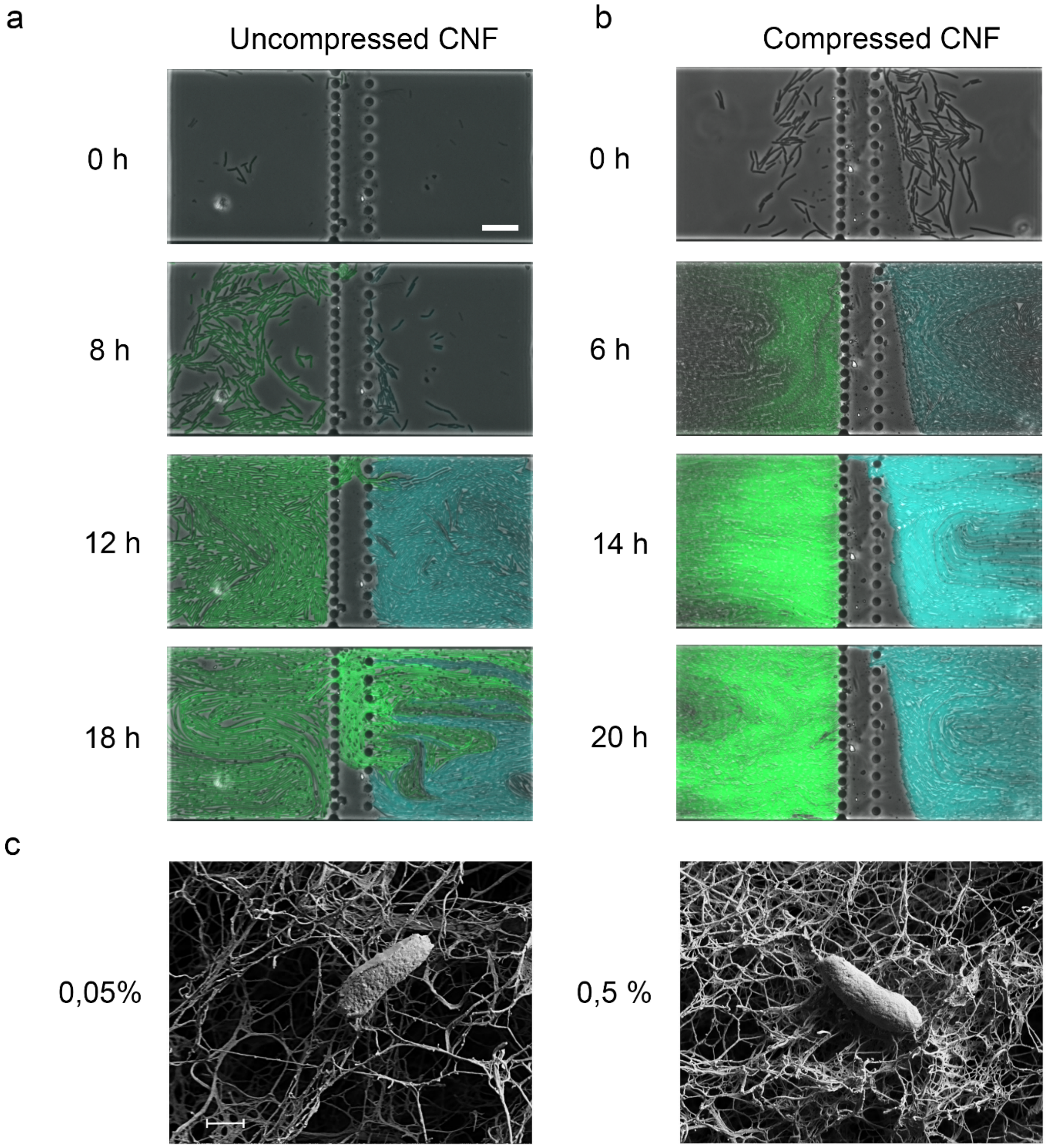

Supplementary Figure 4. Comparison of the CNF filter compression velocity. Phase contrast and fluorescent images of **a**) CNF filters packed with a low velocity (0.13 m s^{-1}) and **b**) high velocity (1.28 m s^{-1}), that both enable diffusion and communication. However, due to the larger pore sizes in the CNF filter at lower packing velocities (**b**), bacterial cells could penetrate after 12 hours and subsequently remove the CNF filter. Scale bar, 20 µm. **c**) SEM images of CNF at 0.05 % w/v and 0.5% w/v respectively. In the 0.05% CNF solution, the pores of the CNF network were large enough for bacterial to enter. Scale bar, 200 nm.

a

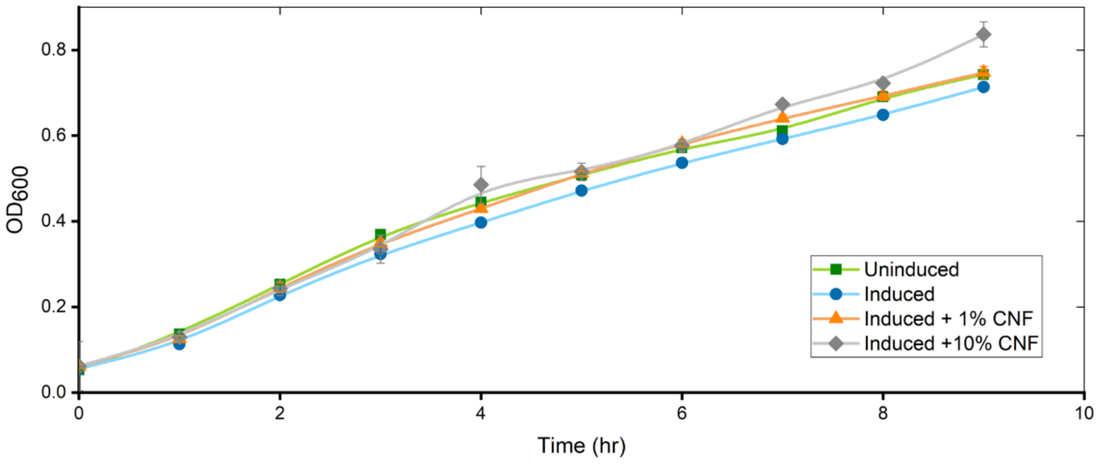

b

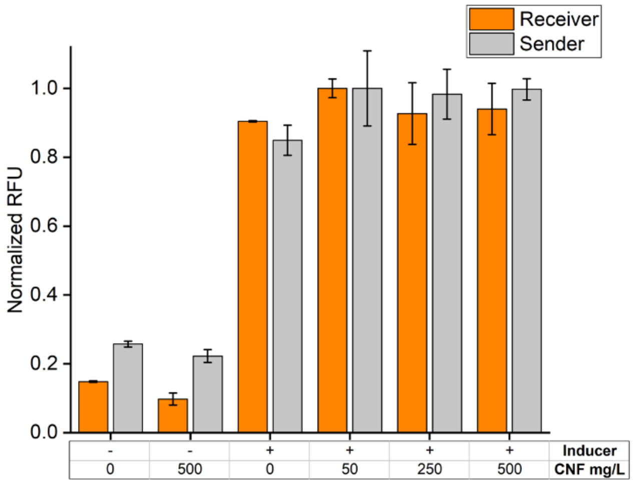

Supplementary Figure 5. Effect of CNF on cellular growth and communication. a) Cellular growth of the receiver population uninduced (■), induced (●) and induced with CNF(▲ and ♦). Both the CNF and inducer had no effect on the overall growth of the cellular population. **b)** Fluorescence intensity of the sender and receiver-populations when cultured with increasing concentrations of CNF after 6 hours of culture time, induced by Arabinose (10 mM) and AHL (10^{-5} M) respectively. The fluorescence intensity appears unaffected by increasing CNF concentrations, both in an AHL producing population (sender) and AHL receiving population (receiver), showing that CNF does not bind AHL and can be used as an efficient filter for cellular separation and communication.

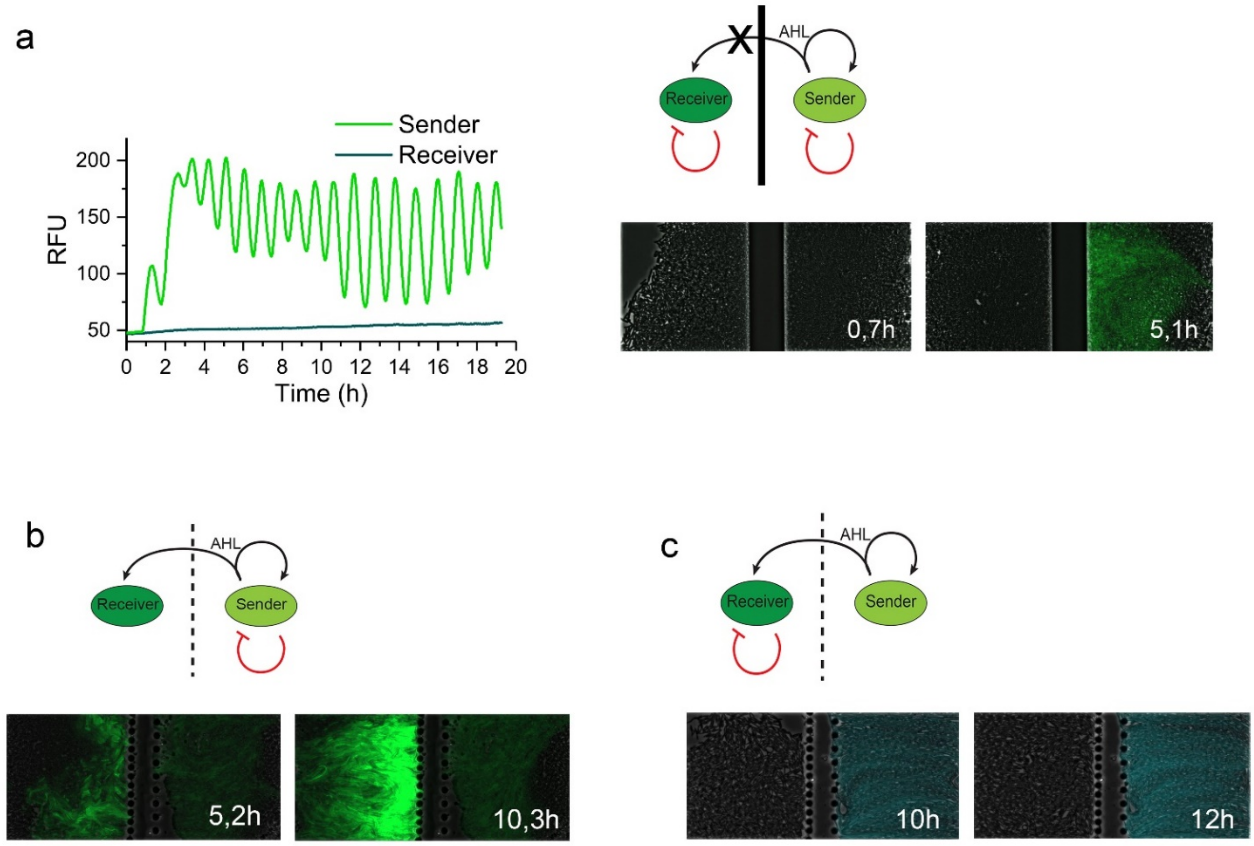

Supplementary Figure 6. Synchronized oscillations in communicating populations. **a**) Phase contrast and fluorescent images of the negative control experiment of Fig. 3a, where a solid PDMS barrier has been placed between the cellular populations, preventing AHL diffusion. This resulted in the absence of fluorescent signal in the receiving population. **b**) Phase contrast and fluorescent images of Fig. 3b, where the fluorescent signal of the sender population oscillates, identical to Fig. 3a and Supplementary Fig. 3a. However, because of the lack of the AiiA lactonase in the receiving population, a very strong fluorescent protein signal accumulates. **c**) Phase contrast and fluorescent images of Fig. 3c, where the absence of AiiA in the sender population results in a stable fluorescent signal. No fluorescent response was detected in the receiving population because of the constant AiiA production, induced by AHL produced in the sender population. Here CFP was used instead of GFP in the sender cells.

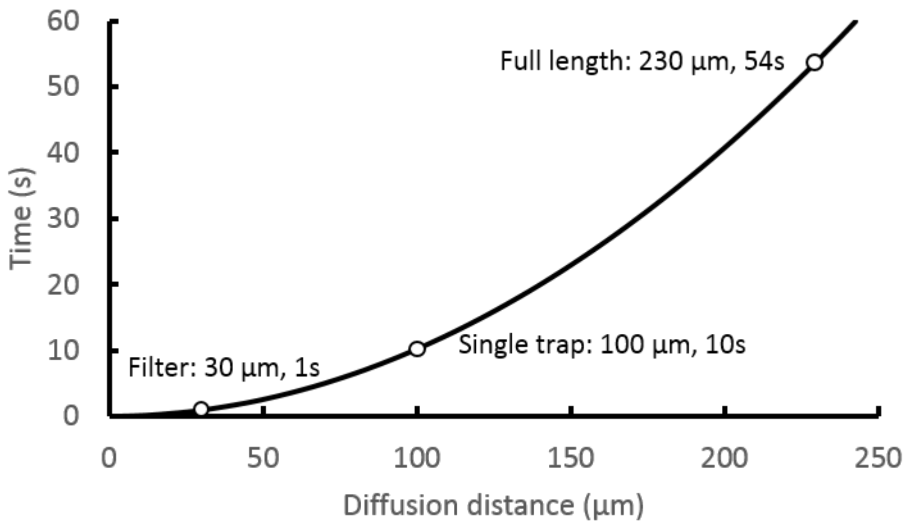

Supplementary Figure 7. AHL diffusion timescales. The time it takes for AHL to diffuse distance x can be approximated by

$$t = \frac{x^2}{2Dt},$$

where t is the typical time needed for a molecule with diffusion coefficient D to diffuse a distance of x in one dimension. For AHL, a diffusion coefficient of $4.9 * 10^{-10}$ m^2 s^{-1} was used. The time it takes for AHL to diffuse across the various distances of the cell communication device are annotated in the figure.

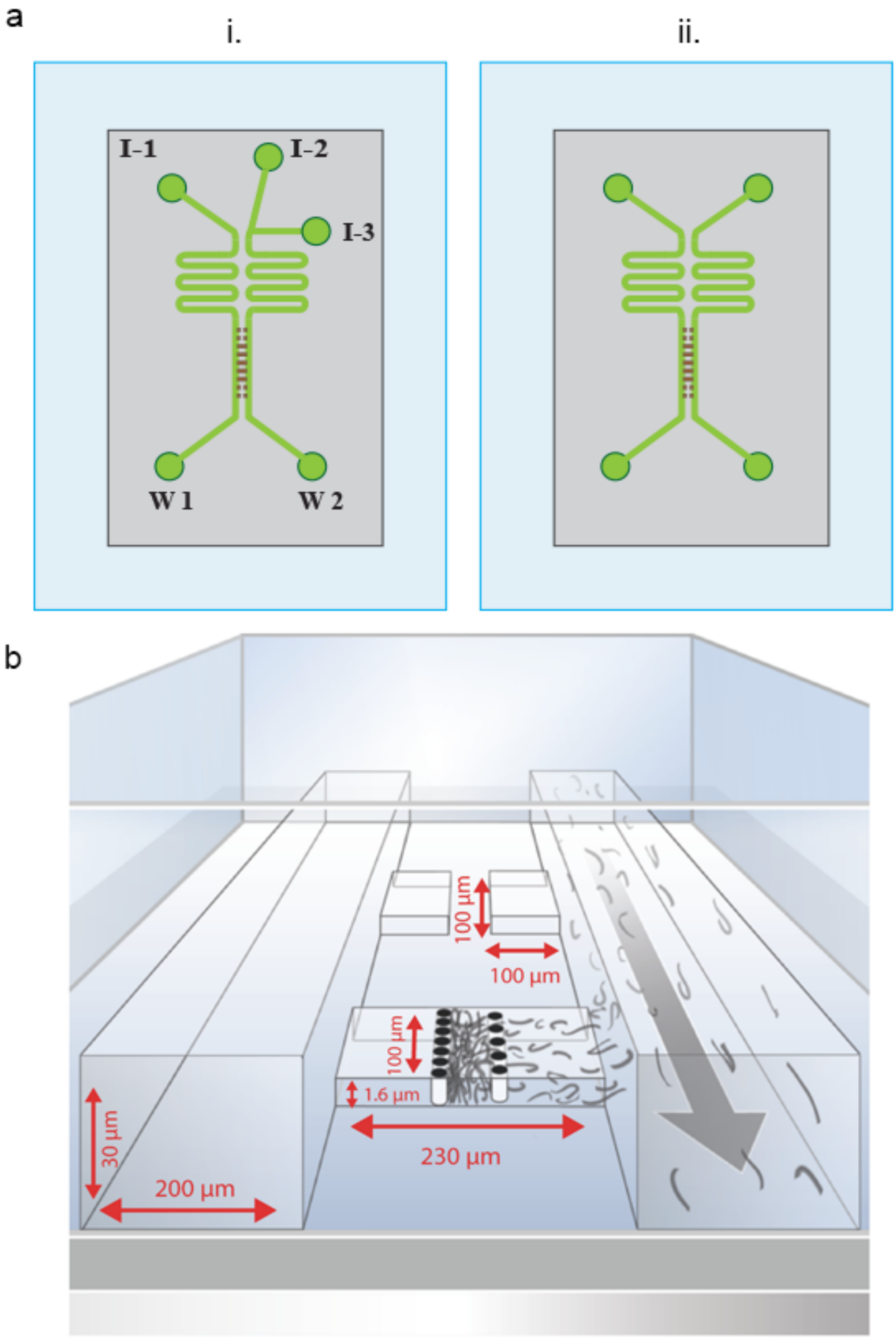

Supplementary Figure 8. Microfluidic chip design. a) Design of the microfluidic chips, indicating the inputs (I) of medium and output/ waste (W). Cells were loaded into the trapping chambers using the wastes, prior to imaging. In stable induction experiments, design i. was used, where I-2 was used to induce the sender population with arabinose added to the medium. In the coupled oscillation experiments, design ii. was used. **b**) Close-up of the microfluidic chip including dimensions, highlighting the connected trapping chambers.
