# Supplementary material for: Controlled communication between physically separated bacterial populations in a microfluidic device
Source: Commun Biol. 2018 Jul 20;1:97. doi: 10.1038/s42003-018-0102-y (PMC6123784; doi:10.1038/s42003-018-0102-y)
Supplement: Supplementary file 2 — Description of Additional Supplementary Files [file 42003_2018_102_MOESM2_ESM.docx]

**Description of Additional Supplementary Files**

File Name: Supplementary Movie 1

Description: A movie showing stable communication between two different cell populations in a trap divided by a cellulose filter in a microfluidic device

File Name: Supplementary Movie 2

Description: A movie showing dynamic oscillating communication between two different cell populations in a trap divided by a cellulose filter in a microfluidic device
